# Chemical Characterization and Bioaccessibility of Bioactive Compounds from Saponin-Rich Extracts and Their Acid-Hydrolysates Obtained from Fenugreek and Quinoa

**DOI:** 10.3390/foods9091159

**Published:** 2020-08-21

**Authors:** Joaquín Navarro del Hierro, Guillermo Reglero, Diana Martin

**Affiliations:** 1 Departamento de Producción y Caracterización de Nuevos Alimentos, Instituto de Investigación en Ciencias de la Alimentación (CIAL) (CSIC–UAM), 28049 Madrid, Spain; joaquin.navarrodel@uam.es (J.N.d.H.); guillermo.reglero@uam.es (G.R.); 2 Sección Departamental de Ciencias de la Alimentación, Facultad de Ciencias, Universidad Autónoma de Madrid, 28049 Madrid, Spain; 3 Imdea-Food Institute, CEI UAM+CSIC, 28049 Madrid, Spain

**Keywords:** fenugreek, quinoa, saponins, diosgenin, oleanolic acid, alkylresorcinols, hydrolysis, in vitro digestion, bioaccessibility, co-excipient foods

## Abstract

Saponin-rich extracts from edible seeds have gained increasing interest and their hydrolysis to sapogenin-rich extracts may be an effective strategy to enhance their potential bioactivity. However, it remains necessary to study the resulting chemical modifications of the extracts after hydrolysis as well as their impact on the subsequent bioaccessibility of bioactive compounds. The chemical composition of non-hydrolyzed and hydrolyzed extracts from fenugreek (FE, HFE) and quinoa (QE, HQE), and the bioaccessibility of saponins, sapogenins and other bioactive compounds after an in vitro gastrointestinal digestion was assessed. In general, FE mainly contained saponins (31%), amino acids (6%) and glycerides (5.9%), followed by carbohydrates (3.4%), fatty acids (FFA) (2.3%), phytosterols (0.8%), tocols (0.1%) and phenolics (0.05%). HFE consisted of FFA (35%), sapogenins (8%) and partial glycerides (7%), and were richer in phytosterols (1.9%) and tocols (0.3%). QE mainly contained glycerides (33%), FFA (19%), carbohydrates (16%) and saponins (7.9%), and to a lesser extent alkylresorcinols (1.8%), phytosterols (1.5%), amino acids (1.1%), tocols (0.5%) and phenolics (0.5%). HQE mainly consisted of FFA (57%), partial glycerides (23%) and sapogenins (5.4%), were richer in phytosterols (2.4%), phenolics (1.2%) and tocols (0.7%) but poorer in alkylresorcinols (1%). After in vitro digestion, saponins from FE and QE were fully bioaccessible, sapogenins from HFE displayed a good bioaccessibility (76%) and the sapogenin from HQE was moderately bioaccesible (38%). Digestion of saponin and sapogenin standards suggested that other components of the extracts were enhancing the bioaccessibility. Other minor bioactive compounds (phytosterols, alkylresorcinols, tocols and some phenolics) also displayed optimal bioaccessibility values (70–100%).

## 1. Introduction

Fenugreek (*Trigonella foenum-graecum* L.) and quinoa (*Chenopodium quinoa* Willd.) are annual crops whose edible seeds have gained special interest in the past decade due to their increasing worldwide production. As an example, fenugreek production in India increased from 116,000 tonnes in 2011 to 247,000 tonnes in 2016, whereas 80,000 tonnes of quinoa were produced worldwide in 2010 and nearly 160,000 tonnes in 2018 [1,2]. While fenugreek is believed to be a legume native of the Eastern Mediterranean region and the Indian subcontinent, the pseudocereal quinoa is an indigenous plant of the Andean region of South America [3,4]. Along with their increased production, the gradual introduction of these seeds in Western cuisine has promoted their growing consumption, to the point where these foods nowadays enjoy great consumer acceptance across Europe, North America and other parts of the world [5,6]. Fenugreek seeds, eaten raw, cooked or sprouted, are bitter to the taste and commonly used as spices in food, as a flavoring in curry powder, infused or as supplements [5]. Quinoa, both sweet and bitter varieties, can be eaten boiled in water and as a rice replacement, popped like popcorn, ground to be used as flour and even sprouted [7]. Together with their corresponding macronutrients, mainly proteins, carbohydrates and lipids, both seeds are sources of minor bioactive phytochemicals, including saponins, phenolic compounds, phytosterols, carotenoids, alkaloids, tocopherols, and they are also rich in essential fatty acids, amino acids, minerals and some vitamins, among many other constituents [8,9]. Due to this composition, current interest in these seeds is focused not only on their culinary and nutritional properties, but also on the obtention of extracts rich in bioactive compounds to be used as nutraceuticals or functional ingredients. 

Within these compounds, saponin-rich extracts from fenugreek and quinoa seeds are of increasing popularity. Saponins are a large group of high molecular weight glycosidic compounds, that consist of a hydrophobic triterpenoid or steroid aglycone, known as sapogenin, attached to one or more hydrophilic sugar moieties through an ether or ester glycosidic linkage [10]. Fenugreek is known for containing steroid saponins, while those from quinoa have been described to be triterpenoid-like. The interest on these bioactive compounds is due to the increasing number of evidences demonstrating their bioactive properties, such as immunostimulatory, hypocholesterolemic, antitumor, antiinflammatory, antibacterial, antiviral, antifungal, and antiparasitic, among others [11,12,13]. 

Concerning the production of saponin-rich extracts, ultrasound-assisted extraction (UAE) has gained special relevance due to significantly reduced energy consumption and extraction times, together with a higher extraction efficiency [14]. The obtention of saponin-rich extracts from fenugreek, quinoa and other seeds by UAE has been effectively performed very recently [15,16,17]. Additionally, as we already demonstrated, other bioactive or interesting compounds can be co-extracted during the production of saponin-rich extracts from fenugreek and quinoa, such as phenolic compounds or lipids [15]. 

In any case, in addition to the own saponin interest *per se*, one of the aims of producing extracts with a relevant saponin content is to further transform them to sapogenin-rich extracts. This is due to the fact that it has been proven that many aglycones exert enhanced bioactivities when compared to their former saponin [18]. In this sense, the most common process described in the literature and also used at industrial scale for the transformation of saponins to sapogenins is the acid hydrolysis [19]. As an example of superior bioactivity of sapogenins, Zhang et al. [20] have recently demonstrated that sapogenins obtained from the acid hydrolyzed products of fenugreek had a greater α-glucosidase inhibitory activity than the saponins as a whole. Similarly, Di Liberto et al. [21] demonstrated that the acid hydrolysate of a saponin-rich extract from *Phytolacca dioica* L. berries showed promising antifungal potency against *Candida albicans* and *Cryptococcus neoformans,* whereas the non-hydrolyzed extract showed low or negligible activity. However, concerning the superior bioactivity of hydrolyzed extracts of saponins, it is interesting to remark that the hydrolysis process itself also leads to the transformation of the whole extracts to yield other bioactive compounds together with sapogenins. Such transformations might also contribute to the whole improved bioactivity. As an example, we recently demonstrated that phytosterols or tocopherols were also increased together with sapogenins after the acid hydrolysis of fenugreek and quinoa extracts [22]. Therefore, a further deep chemical characterization of hydrolyzed saponin-rich extracts would be of great interest in order to understand the behavior and transformation of certain other components when submitted to hydrolysis. The characterization of both extracts may allow a complete understanding of the subsequent bioactivity. 

Regardless of the effect of the hydrolysis process on the composition of the extracts, their bioactivity is closely related to their intestinal bioaccessibility [18]. This term refers to the amount of a compound that is released from its matrix in the gastrointestinal tract and thus becomes available for intestinal absorption [23]. In other words, it may be defined as the fraction of a total ingested given compound that remains solubilized and stable before cell absorption. In the specific case of saponins from different seed extracts, such as fenugreek, quinoa, lentil, soybean or lupin, a wide range of bioaccessibilities has been recently described [15]. These values varied between 13 and 83% for aqueous ethanol extracts, although the bioaccessibility of saponins contained in ethanolic extracts was almost complete in all cases. Several factors were related to such variability, being the co-extracted compounds a relevant one. Thus, it was found that the total phenolic content limited the bioaccessibility of saponins, whereas the fat content or the saponin/fat content ratio enhanced this parameter [15]. Hence, it is important to bear in mind that the characterization of co-existing compounds in the extracts and the study of their bioaccessibility may help to understand the whole gastrointestinal behavior of the extracts and their further potential bioactivity.

While the available literature regarding the bioaccessibility of saponins is relatively scarce, the studies focusing on the bioaccessibility of sapogenins are, to the best of our knowledge, even more limited than for saponins [18,24,25]. Nevertheless, the bioavailability of sapogenins has been more deeply studied and very low values have been described. This suggests that the poor intestinal absorption of aglycones is consequence of a relatively poor bioaccessibility, due to their low aqueous solubility as non-polar compounds [26]. It is important to remark, however, that the poor bioaccessibility expected for sapogenins may be linked to the aglycones when consumed alone. This is because certain components of the extracts or the co-digestion with certain food excipients of lipid nature may be considerably enhancing the solubility of these compounds in the intestinal tract, and hence their bioaccessibility [25,27]. Therefore, the study of the bioaccessibility of sapogenins as part of a whole extract would be of interest before to generally stating that these compounds would have a poor bioaccessibility. This same hypothesis may be considered for the bioaccessibility of other bioactive compounds contained in the hydrolyzed saponin-rich extracts, such as phytosterols, tocols or phenolic compounds, among others. 

The aims of this study were: (1) to tentatively study the modification of the chemical composition of saponin-rich extracts obtained from fenugreek and quinoa seeds after their acid hydrolysis, analyzing changes in the saponin and sapogenin content, as well as the modification in the composition of other co-existing compounds in the extracts; and (2) to assess the bioaccessibility of saponins, sapogenins and other co-existing bioactive compounds of the extracts after the in vitro simulation of the gastrointestinal digestion. Furthermore, the bioaccessibility of individual saponin and sapogenin standards, namely dioscin, diosgenin and oleanolic acid, was also evaluated for comparative purposes with the extracts.

## 2. Materials and Methods 

### 2.1. Reagents and Materials

Commercial seeds of fenugreek (*Trigonella foenum-graecum* L.) were purchased from Murciana de Herboristeria (Murcia, Spain) and seeds of red quinoa (*Chenopodium quinoa* Willd.) were purchased from Hijo de Macario Marcos (Salamanca, Spain). Trizma base, maleic acid, sodium chloride, calcium chloride, HCl, Amano lipase A from *Aspergillus niger*, pepsin, pancreatin from porcine pancreas, bile salts, phosphatidyl choline from egg yolk, β-sitosterol (≥70%), oleic acid, lysine, 1,3-diolein, 1-oleoyl-rac-glycerol, *myo*-inositol, d-glucose, quercetin, diosgenin, hederagenin, protocatechuic acid, squalane, and N,O-bis-(trimethylsilyl)trifluoroacetamide (BSTFA) were from Sigma-Aldrich Chemie GmbH (Steinheim, Germany). Sucrose was from Panreac (Barcelona, Spain). Dioscin, protodioscin, 5-pentadecylresorcinol and oleanolic acid were from Cymit Quimica S.L (Barcelona, Spain). Methanol, hexane, 1-butanol, chloroform were from Macron (Gliwice, Poland).

### 2.2. Obtention of the Saponin-Rich Extracts by Ultrasound-Assisted Extraction (UAE)

Seeds were ground in a knife mill (Grindomix GM200, Retsch, Haan, Germany) at 10,000 rpm for 1 min and the resulting powder was sieved in a vertical sieve (CISA Cedacería Industrial, Barcelona, Spain) until obtaining fractions with a particle size between >100 µm and ≤250. The subsequent extraction was based on Navarro del Hierro et al. [27]. Samples were extracted with methanol at a ratio of sample to solvent of 1:10 (w/v) for 15 min by direct sonication (Branson SFX250 Digital Sonifier, Branson Ultrasonics, Danbury, CT, USA) with an ultrasonic probe (1/2” diameter, output sonication amplitude of 60%) at 20 kHz in a glass vial. Then, the mixture was centrifuged at 3400× *g* for 15 min. The supernatant was defatted with hexane at a ratio of 1:1 (v/v) by vortex agitation for 1 min and centrifuged at 2688× *g* for 10 min. The top hexane phase was removed with a pipette and the methanolic phase was collected and rotary evaporated under vacuum (Valor Hei-VAP, Heidolph Instruments, Schwabach, Germany). In order to achieve a further enrichment in the compounds of interest, the dried residue was extracted with water and 1-butanol in Falcon tubes. Briefly, milliQ water was added to the dried residue at a ratio of sample to solvent of 1:20 (w/v). Once solubilized, 1-butanol was added to the mixture at a ratio of water to 1-butanol of 1:2 (v/v), vortexed for 1 min and centrifuged at 2688× *g* for 10 min. The top phase was collected and the bottom phase was extracted again under the same proportions and conditions. Both collected phases were dried under vacuum, extraction yield (EY) was estimated and the resulting extracts (fenugreek extract, FE; quinoa extract, QE) were stored at −20 °C until further use. This process was performed at least in quintuplicate.

### 2.3. Preparation of the Sapogenin-Rich Extracts

The previously obtained saponin-rich extracts were mixed to obtain a single extract to be acid-hydrolyzed as described by Navarro del Hierro et al. [27]. Briefly, the saponin-rich extracts were heated at 100 °C (Stuart™ Block Heater, Cole-Parmer, Staffordshire, UK) with HCl solution (2 mol L^−1^) at a ratio of sample to acid solution of 1:50 (w/v) for 1 h in glass vials. After, the mixture was ice-cooled for 5 min and liquid-liquid extracted with ethyl acetate at a ratio of 1:1 (v/v) by vortex agitation for 1 min and centrifuged at 3400× *g* for 5 min. The top phase was collected and the bottom phase was extracted again with the same volume of ethyl acetate under the described conditions. Both collected phases were dried under N_2_ stream, EY was estimated and the resulting extracts (hydrolyzed fenugreek extract, HFE; hydrolyzed quinoa extract, HQE) were stored at −20 °C until further use. This procedure was performed at least in triplicate and once the EY was estimated, all replicates were mixed to obtain a single extract. 

### 2.4. Characterization of the Extracts by High Performance Liquid Chromatography-Diode Array Detection

Saponins were tentatively characterized on a LC-2030C 3D Plus system (Shimadzu, Kyoto, Japan), following the method described by Herrera et al. [22]. The system was equipped with a quaternary pump and a DAD. Separation of compounds was carried out on an ACE 3 C18-AR column (150 mm × 4.6 mm, 3 μm particle size) protected by a guard column (Advanced Chromatography Technologies Ltd, Aberdeen, Scotland). A gradient elution was performed using water with 0.05% TFA (phase A), and acetonitrile with 0.05% TFA (phase B). The method was as follows: 0–20 min, 95–5% A; 20–45 min: 5% A; 45–46 min: 5–95% A; 46–50 min 95% A. The flow rate was constant at 0.4 mL/min, and the column temperature was kept at 25 °C. The injection volume was 20 μL, and UV-Visible spectra were recorded from 190 to 700 nm, whereas the chromatograms were registered at 210 nm. Quantification of saponins was performed by calibration curves obtained from hederacoside C for saponins from quinoa and protodioscin for saponins from fenugreek. Both calibration curves were prepared at 0.12, 0.24, 0.48, 0.72 and 0.97 mg/mL. 

### 2.5. Characterization of the Extracts by Gas Chromatography-Mass Spectrometry

Prior to characterization, 10 mg of sample was derivatized with 1 mL of BSTFA by heating at 75 °C for 1 h in a block heater (Stuart™ Block Heater). Then, extracts were tentatively characterized according to Herrera et al. [22], the derivatized samples were analyzed by GC-MS (Agilent 7890A, Agilent Technologies, Santa Clara, CA, USA) comprising a split/splitless injector, an electronic pressure control, a G4513A autoinjector, and a 5975C triple-axis mass spectrometer detector. The column used was an Agilent HP-5MS capillary column (30 m × 0.25 mm i.d., 0.25 μm phase thickness). Helium was used as carrier gas at 2 mL/min. The injector temperature was 260 °C, and the mass spectrometer ion source and interface temperatures were 230 and 280 °C, respectively. The sample injections (1 μL) were performed in splitless mode. The oven temperature was initiated at 50 °C, held for 3 min and increased at a rate of 15 °C/min to 310 °C, then held for 25 min. The mass spectra were obtained by electronic impact at 70 eV. The scan rate was 1.6 scans/s at a mass range of 30–700 amu. Identification of compounds was performed by the NIST MS Data library, the mass spectra according to literature, or according to those of pure commercial compounds whenever possible. Quantification of compounds was performed by calibration curves obtained from commercial standards whenever possible. The commercial standards oleanolic acid and hederagenin were used for the quantification of sapogenins from quinoa, while diosgenin was used for the quantification of those from fenugreek. For other additional compounds of the extracts, oleic acid was used for the quantification of FFA, 1,3-diolein was used for diacylglycerides (DAG), 1-oleoyl-*rac*-glycerol was used for monoacylglycerides (MAG), lysine was used for amino acids, d-glucose was used for monosaccharides, *myo*-inositol was used for sugar alcohols, sucrose was used for disaccharides, protocatheccuic acid was used for phenolic compounds, quercetin was used for itself, 5-pentadecylresorcinol was used for alkylresorcinols, β-sitosterol was used for the quantification of both phytosterols and tocopherol, and finally squalane was used for squalene. 

### 2.6. In Vitro Gastrointestinal Digestion

The in vitro digestion model was based on Navarro del Hierro et al. [15] with slight modifications. In this study, the whole process of digestion was performed in 50 mL Falcon tubes to allow the direct centrifugation at the end of digestion for the bioaccessibility determination, as explained in Section 2.7. For gastric digestion, 1.1 mL of a gastric solution at pH 2.5 (150 mM NaCl, 6 mM CaCl_2_ and 0.1 mM HCl) were added to 12.5 mg of the extracts, to reach a concentration of 5 mg/mL at the end of the digestion. For the assays with commercial standards and for comparative purposes, the initial digested amount was equal to the amount of saponins or sapogenins contained in 5 mg/mL of either FE, HFE or HQE at the end of the digestion, that is, 3.9 mg for dioscin, 1 mg for diosgenin and 0.70 mg for oleanolic acid, respectively. Then, the mixture was gently stirred at 250 rpm in an orbital incubator (Titramax 1000 package, 177 Heidolph Instruments) at 37 °C for 1 min to allow the dispersion of the components. The gastric digestion started after the addition of 0.225 mL of a fresh extract of gastric enzymes containing gastric lipase (16 mg/mL) and pepsin (5 mg/mL) in gastric solution previously stirred for 10 min. The gastric digestion was performed for 45 min and 250 rpm. Then, for the intestinal digestion, 0.95 mL of a solution simulating a biliary secretion (50 mg of lecithin and 125 mg of bile salts in 0.25 mL of 325 mM CaCl_2_ solution, 0.75 mL of 3.25 M NaCl solution, and 5 mL of Trizma-maleate buffer 100 mM pH 7.5) were added. After the addition of the biliary secretion, the whole medium was stirred for 1 min at 37 °C. The intestinal digestion was initiated by the addition of 0.225 mL of the supernatant of a fresh pancreatin extract at 15.6 mg/mL in trizma-maleate buffer, which had been previously stirred for 10 min and centrifuged at 2688× *g* for 15 min. The reaction was performed for 60 min at 250 rpm. Digestions were prepared at least in duplicate. 

### 2.7. Determination of the Bioaccessibility of Bioactive Compounds

For the determination of the bioaccessibility of saponins, the digestion medium was submitted to centrifugation at 2688 × g for 40 min in order to obtain the micellar phase. This phase contained the solubilized or bioaccessible saponins. Hence, aliquots from both the digestion medium and the micellar phase were directly analyzed as described in Section 2.4 by HPLC-DAD, but in this case, the chromatograms were registered at 317 nm (for fenugreek saponins) and 323 nm (for quinoa saponins), to avoid the interference of the chromatographic signal derived from the digestion medium.

For the determination of the bioaccessibility of sapogenins and the rest of bioactive compounds, two sets of digestions were prepared in duplicate. One, which was the whole digestion medium and the second one, the isolated micellar phase. Then, according to Navarro del Hierro et al. [27], each of the digestions were vortex-extracted for 1 min with ethyl acetate at a ratio of 1:1 and centrifuged for 10 min at 2688× *g*. The top phase was collected and the bottom phase was extracted again with the same volume of ethyl acetate under the described conditions. Both collected phases were dried under N_2_ and the resulting residue was derivatized and analyzed by GC-MS as described in Section 2.5.

The bioaccessibility of all compounds was calculated as follows:

Bioaccessibility (%) = (*weight of compound in micellar phase*/x*weight of compound in digestion medium*) × 100

### 2.8. Statistical Analysis

Statistical analyses were performed by means of the general linear model procedure of the SPSS 26.0 statistical package (SPSS Inc., Chicago, IL, USA) by one-way analysis of variance. Differences were considered significant at *p* ≤ 0.05.

## 3. Results & Discussion

### 3.1. Extraction Yield

The first parameter analyzed prior to the characterization of each of the extracts was the EY, defined as the weight of crude dried extract obtained respect to the weight of ground seed used for extraction, expressed as percentage. In general, the UAE extraction using methanol and the further concentration with butanol in order to obtain extracts with an increased saponin content led to differences in the EY between the two studied seeds. The EY of the FE was 5.57 ± 0.59%, whereas that from QE was 2.12 ± 0.10% (*p* < 0.001). These values, and specially that from quinoa, are considerably lower than the ones previously described for ethanolic extracts from these same seeds (9.35% for fenugreek and 14.8% for quinoa), likely due to the fact that such extracts were obtained with a less polar solvent, were not deffated and were not further concentrated with butanol, but these operations did not favor a higher enrichment in saponins [15]. 

When the acid hydrolysis was performed for the obtention of sapogenin-rich extracts, differences between the two types of extracts (non-hydrolyzed/hydrolyzed) and the two seeds were also observed (*p* < 0.001). Specifically, the EY of the HFE was 2.90 ± 0.08% and that from HQE was 1.41 ± 0.04%, which was similar to the 0.9% EY recently described for an acid-hydrolyzed *Hedera helix* L. extract rich in hederagenin, a triterpenoid aglycone also occurring in quinoa seeds [28]. 

Therefore, from the EY point of view, and taking into account the economical relevance of this parameter, the fenugreek seeds are more interesting than quinoa seeds to obtain either saponin-rich extracts and sapogenin-rich extracts.

### 3.2. Saponin and Sapogenin Content on the Extracts

The saponin and sapogenin content of the non-hydrolyzed and hydrolyzed extracts from fenugreek and quinoa is shown in Table 1 and their chromatograms are shown as Figures S1 and S2. It should be noted that the inadequate baseline separation of some saponin peaks may lead to inaccurate quantitation values of some individual saponins, although the inadequate separation of saponins from extracts is a widespread issue that should be taken into consideration. Therefore, a tentative estimation on saponin content of the extracts was performed. Focusing first on the non-hydrolyzed extracts, steroidal saponins in the FE were the major identified compounds with a content around 30%, whereas triterpenoid saponins in the QE accounted for around 8% of the extract (Table 1). For more detailed information about the chemical structure of these saponins, we recommend our previous study in which most of the saponins contained and characterized in these extracts were tentatively identified for the first time in similar extracts by HPLC-MS-DAD [22]. 

After the acid hydrolysis of the extracts, a similar residual content of saponins was found in both HFE and HQE (being around 2%), which might be due to the time of reaction not being enough for the complete release of aglycones from the sugar moieties (Table 1). However, it should be considered that the optimal time of hydrolysis in which the maximum amount of sapogenins is guaranteed for these extracts was 1 hour, since longer times of hydrolysis caused a reduction in the sapogenin content due to a degradation of aglycones simultaneously with their release, as we already demonstrated [22]. Further studies assessing the use of less aggressive and more environmentally friendly acids that guarantee the complete hydrolysis of saponins without causing a negative impact on the final sapogenin content might be of interest. 

Concerning sapogenins, the deglycosylation of saponins led to a HFE with a sapogenin content of around 8% (Table 1). Among the identified steroidal sapogenins, diosgenin was the major aglycone (close to 3%), followed by its epimer yamogenin and gitogenin. Sarsasapogenin, tigogenin and its 25*S* isomer neotigogenin were found in very similar concentrations each. The aglycones neogitogenin, smilagenin and yuccagenin were the least concentrated ones. Two spirostadienes were also identified and they accounted for only around 5% of the total sapogenin profile of the HFE. These last compounds are artefacts generated by dehydration reactions of diosgenin and yamogenin in presence of hydrochloric acid [29]. 

The sapogenin content of the HQE was close to 5% (Table 1). The main four identified aglycones, in decreasing order of content, were serjanic acid, oleanolic acid, phytolaccagenic acid and hederagenin. This same sapogenin profile has been recently described for a large number of quinoa varieties, the sapogenin content widely varying between samples [30]. In addition, the presence of phytolaccagenic acid may be an indicator of bitter quinoa cultivars, as it has been suggested that sweet varieties have no detectable amounts of this aglycone [31]. 

Therefore, fenugreek seeds seem to be more interesting than quinoa seeds from the point of view of a higher enrichment in saponins after extraction and sapogenins after the hydrolysis of such extracts. Nevertheless, the hydrolyzed extracts from fenugreek and quinoa would be of interest as rich sources of different types of sapogenins, namely steroid and triterpenoid sapogenins, whose bioactive interest might also be different. Further studies considering a higher number of replicates of extracts would be of interest in order to confirm these evidences.

### 3.3. Characterization of Other Compounds in the Extracts

**Figure 1:**
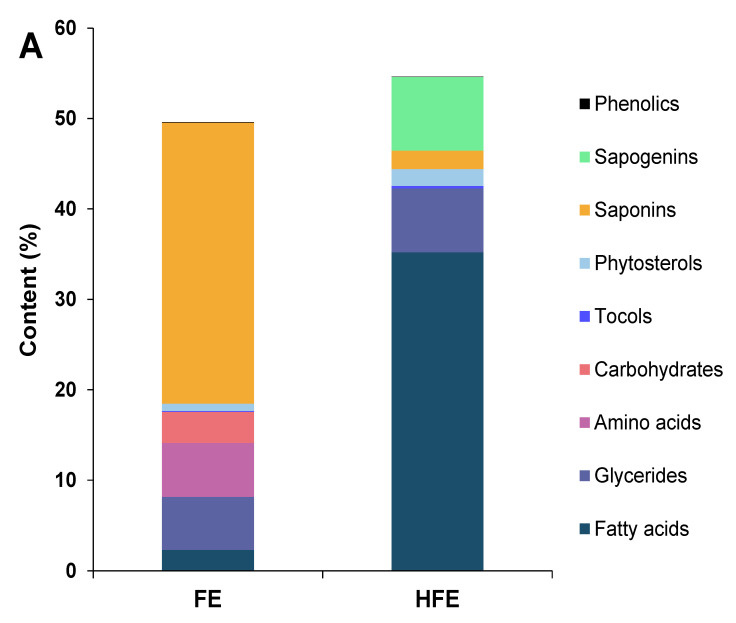

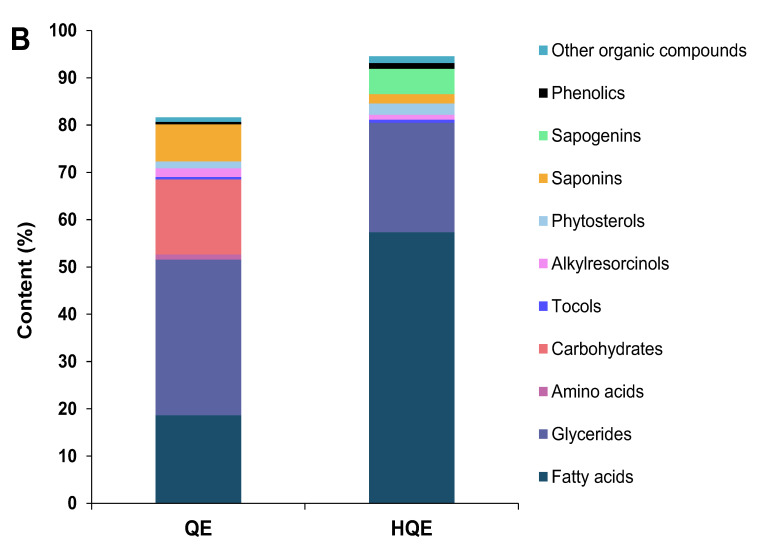
Concentration (%) of each characterized family of compounds from (**A**) non-hydrolyzed (FE) and hydrolyzed extracts (HFE) from fenugreek; and (**B**) non-hydrolyzed (QE) and hydrolyzed (HQE) extracts from quinoa.

A general and preliminary characterization of the four different extracts from fenugreek and quinoa was performed by GC-MS previous formation of trimethylsilyl derivatives of all those less volatile compounds containing carboxyl or hydroxyl functional groups. This method allowed to tentatively identify up to 27 compounds in the FE, 33 compounds in the HFE, 80 compounds in the QE and 66 compounds in the HQE different to saponins and sapogenins (Table 2 and Table 3). Compounds were categorized into 12 (for fenugreek) or 13 (for quinoa) sub-groups depending on their principal chemical family, being mainly lipids (fatty acids and glycerides), nitrogen compounds (amino acids and derivatives and non-protein nitrogen compounds), phenolic compounds, organic compounds, carbohydrates and derivatives (sugars and sugar alcohols), tocols, physterols, other organic acids and alkylresorcinols (only in quinoa). In addition to Table 2 and Table 3, in order to enhance the comparison between the non-hydrolyzed and the hydrolyzed extracts of each seed, and to show a general illustration of the complex composition of the extracts, the total content of each major chemical group, including saponins and sapogenins, from fenugreek and quinoa is shown in Figure 1A,B, respectively.

#### 3.3.1. Fenugreek Extracts

First, regarding the total amount of quantitated compounds in each of the extracts, around 50% of the FE was tentatively characterized and quantitated, while in the case of the HFE, such value slightly increased up to a 54%. 

While in the FE the majority of the extract corresponded to saponins (around 30%), nearly 35% of the HFE corresponded to FFA (Figure 1A), whereas FFA accounted for only 2% in the FE before hydrolysis. This relevant increase in FFA would be mainly due to their release from residual lipids of the extracts during the acid hydrolysis. In this sense, the total initial triacylglycerides (TAG) content for FE was close to 6%, as already reported [27], being also included in Figure 1A under glycerides. Among the FFA, linoleic and oleic acids were the most abundant ones in both extracts (Table 2). Other FFA in the HFE were the palmitic and stearic acids. Other interesting health-related FFA found in the HFE were α-linolenic and azelaic acids.

The hydrolysis of lipids from the extracts after acid reaction to yield FFA could also explain the presence of partial glycerides, as DAG and MAG in case of the HFE (Table 2). The DAG accounted for the majority of partial glycerides (6%), while the most abundant MAG was 1-monoolein. Partial glycerides have widely proven their beneficial effects as emulsifiers and absorption enhancers, very important aspects to be considered for the subsequent intestinal absorption of lipophilic bioactive compounds such as sapogenins in the case of sapogenin-rich extracts [32]. 

Therefore, considering these results, it is important to remark that the hydrolysis process of saponin-rich extracts from fenugreek and the subsequent purification to extract sapogenins to produce a sapogenin-rich extract, seems to lead to a final extract especially rich in lipids under the form of FFA and partial glycerides. This might be explained by the hydrolysis of residual lipids and the later purification process of sapogenins with ethyl acetate, causing a selective extraction of non-polar compounds of the hydrolysed saponin-rich extract. 

This purification process by ethyl acetate might also explain why other macronutrients such as amino acids and sugars were only found in the FE (Table 2 and Figure 1A), as the extraction with the organic solvent after the acid hydrolysis caused the more polar compounds to remain in the aqueous phase of the reaction medium. Concerning these more polar compounds of the FE, the major identified amino acid was glutamic acid (nearly 5%) and three other structurally similar derivatives, followed by phenylalanine, l-tyrosine and l-valine (Table 2). This amino acid profile in the FE is in accordance with previous determinations of the amino acid composition of fenugreek seeds, especially considering the high abundance of glutamic acid [33]. On the other hand, the major detected carbohydrates were sugar alcohols (close to 2%), followed by sucrose and other disaccharides. Despite most of these carbohydrates could not be clearly identified by the used analytical procedure, very recently, Lahuta et al. [34] found that the most abundant sugar alcohols in fenugreek seeds were d-pinitol and α-d-galactosides of d-pinitol, as well as galactosides of *myo*-inositol. 

Regarding the presence of bioactive compounds other than saponins and sapogenins, the acid hydrolysis of the FE caused interesting differences between both extracts. The concentration of identified phytosterols increased by more than two folds in the HFE with respect to the FE and a similar result was observed for the concentration of α-tocopherol (Table 2). In addition, β-hydroxy-β-methylglutaric acid, or meglutol, known for its hypolipidemic properties and commonly found as a complexed compound in lignans from flaxseeds [35,36], was only detected in the HFE (Table 2). Its presence in fenugreek has not been described before, however, considering that this compound may be conjugated with phenolic compounds [37], its appearance in the HFE could be due to its release after the hydrolysis. 

Only two phenolic compounds were detected by GC in the fenugreek extracts. While 4-hydroxybenzoic acid was found only in the HFE, a non-identified phenolic compound was detected in the FE at a similar concentration (Table 2). Phenolic compounds from fenugreek have been described to be acylated and non-acylated flavonoids with apigenin, luteolin and kaempferol as aglycons [38]. The high molecular weight of these compounds do not make GC the most suitable tool for their analysis, hence further studies about the phenolic composition of the extracts by other analytical techniques might be worth to be performed. 

Finally, it was also detected the presence of 5-(hydroxymethyl)furfural (HMF) only in the HFE, a compound which likely resulted from the Maillard and caramelization reactions during the acid hydrolysis of the saponin-rich extracts, probably due to the amino acid and carbohydrate content. Even though the toxicity of HMF has been generally assumed and its daily intake has been well established, controversial conclusions on the biological effects of this furanic compound have been drawn, as its antioxidant, antiproliferative and antiischemic activities have been confirmed [39]. 

Therefore, as summary, and considering the sum of all the remarked bioactive compounds of the extracts different to saponins and sapogenins (tocopherol, phytosterol and phenolic compounds), this general and preliminary characterization shows that the hydrolysis process of FE seems to lead to a relevant enrichment of these compounds, varying from around 1% of the FE to around 2% for the HFE, in addition to the 8% sapogenin content. However, it would be necessary to carry out more analysis of a greater number of replicates of extracts to confirm these evidences.

#### 3.3.2. Quinoa Extracts

In terms of the total amount of quantitated compounds in each of the extracts, nearly 82% of the QE was tentatively characterized and quantitated, while in the case of the HQE, such value considerably increased to nearly 95% (Figure 1B). 

The most abundant family of compounds in both extracts were lipids, although while in the QE the glycerides (TAG, DAG and MAG) accounted for the major subgroup (around 30%), followed by FFA (close to 20%), in the HQE nearly 60% of the extract accounted for FFA and around 20% for partial glycerides (DAG and MAG), suggesting the hydrolysis of lipids after the acid treatment of the QE, similar to what occurred in the FE. Linoleic and oleic acids were the most abundant FFA in both QE and HQE, followed by palmitic acid, whose content increased by more than four folds in the HQE (Table 3). It is worth mentioning the presence of palmitoleic acid in HQE, possibly a product of hydrolysis given its inexistence in the QE. Omega-7 monounsaturated fatty acid has been reported to have beneficial effects on insulin sensitivity, cholesterol metabolism, hemostasis and thrombosis prevention [40]. Regarding the glyceride composition, only three MAG were identified in the QE (total content of around 2%), while seven MAG with a concentration close to 5% were detected in the HQE. The major one in this last extract was again 1-monoolein and its concentration was nearly five times higher than in the HFE. Regarding the rest of glycerides, DAG were found in a similar concentration in both QE and HQE (around 20%) and TAG accounted for around 9% of the QE, as previously described [27].

Therefore, like for fenugreek, the hydrolysis process of saponin-rich extracts from quinoa and the subsequent purification to extract sapogenins to produce a sapogenin-rich extract, seems to lead to a final extract especially rich in lipids under the form of FFA and partial glycerides, due to the purification process of sapogenins by ethyl acetate. 

On the other hand, likewise fenugreek extracts, sugars and amino acids were only found in the QE. The carbohydrate fraction of the QE was the second largest group after lipids, accounting for nearly 16% of the extract, around 80% of which corresponded to sucrose (Figure 1B and Table 3). The rest of the compounds were mostly non-identified monosaccharides and sugar alcohols. Other authors have recently confirmed that sucrose is the major sugar in quinoa flours, including also the presence of glucose, fructose and *myo*-inositol phosphates [41,42].

The major identified amino acid was l-tyrosine, followed by four essential amino acids (l-valine, l-leucine, l-isoleucine and phenylalanine) with a similar content (Table 3). These results are in agreement with the essential amino acid composition of quinoa seeds, as it has been reported that tyrosine and phenylalanine are the most abundant ones and the content of the rest of amino acids here identified is also in a similar equivalent range [43]. 

In terms of the composition of bioactive compounds different to saponins and sapogenins, very interestingly, the pentacyclic triterpene β-amyrin was also detected in both QE and HQE (Table 3), confirming the fact that free aglycones (non glycosylated) may also be occurring in the seeds, although its content could not be determined due to co-elution with another compound. The isolation of this triterpene from the seeds of quinoa and others such as α-amyrin, echinocystic acid and erythrodiol has previously been described, with associated biological activities such as antibacterial, antioxidant and antiproliferative [44]. Regarding the presence of other bioactive compounds, three phytosterols with a total content lower than 2% were identified in the QE (Table 3). 

In contrast to fenugreek, between seven and eight phenolic compounds were detected in both QE and HQE, respectively (Table 3). The enrichment observed in the hydrolyzed extract was mainly due to the concentration effect of vanillic acid and quercetin, and the appearance of a non-identified phenolic compound. All the phenolics detected have been previously confirmed to occur in quinoa seeds [46,47]. In any case, the analysis of QE and HQE by other analytical techniques might also be of interest in order to precisely determine the phenolic profile of the extracts. 

Regarding the presence of other organic compounds, the HQE also contained HMF, although it should be noted that, considering its chromatographic area, its presence in the HQE was more than 5 times higher than in the HFE, possibly due to the considerably richer content in carbohydrates that may interact with aminoacids during the hydrolysis process. The unsaturated open-chain triterpene squalene was also detected in both quinoa extracts, although its concentration in the HQE was nearly 1.5 times higher than in the QE. 

Lastly, 5-n alkylresorcinols (ARs), which are a very interesting and novel family of long-chain phenolic lipids, were identified in both extracts (Table 3). Specifically, even and odd ARs, methylalkylresorcinols (MARs) and branched-chain alkylresorcinols (bcARs) were detected. Their content in the HQE was lower than in the QE and the concentration of these compounds was lower than 1%. It is worth highlighting that, as far as we are concerned, the identification in quinoa of unsaturated ARs has not been reported in the literature before, although Ross et al. [45] did identify unsaturated MARs, together with an exhaustive characterization of other phenolic lipids. In our work, the novel identified unsaturated ARs were C21:1 (*m/z* 268, M^+^· 546), C22:1 (*m/z* 268, M^+^· 560) and C23:1 (*m/z* 268, M^+^· 574). The relevance of phenolic lipids lies in the fact that even-numbered ARs seem to be exclusively found in this seed, hence being useful as biomarkers of quinoa intake [45]. Despite their novelty, multiple bioactivities linked to ARs have been already described, such as antitumor, antibacterial, antioxidant and inhibition of relevant enzymes [48]. As a contribution of the current study to the field of ARs, it is shown that saponin and sapogenin-rich extracts of quinoa might be interesting sources of ARs, but these compounds seem to be labile to the acid hydrolysis processes, taking into account the observed decrease in the sapogenin-rich extract.

Therefore, considering the sum of all the bioactive compounds of the extracts different to saponins and sapogenins (tocopherol, phytosterols, phenolic compounds, squalene, and alkylresorcinols), this general and preliminary characterization shows that the hydrolysis process of QE seems to lead to a slight enrichment, varying from around 5% of the QE to around 7% for the HQE, in addition to the sapogenin content close to 6%. Compared to fenugreek, the diversity and amount of these bioactive compounds was superior for quinoa, although it should be considered that nearly half of the fenugreek extracts remain uncharacterized. In any case, the total sum of bioactive compounds in the hydrolyzed extracts from both seeds, including sapogenins, was similar (10% for fenugreek and 12% for quinoa). Further studies considering a more exhaustive characterization, and assessing the relevance of this modified composition in bioactive compounds after the acid treatment on different biological activities would be of interest. Additionally, given that a single quantitation of the extracts has been performed from a mixture of different extractions and hydrolysis, a larger number of replicates to be further quantitated should be considered in future works. 

### 3.4. Bioaccessibility of Bioactive Compounds

#### 3.4.1. Saponins

**Figure 2:**
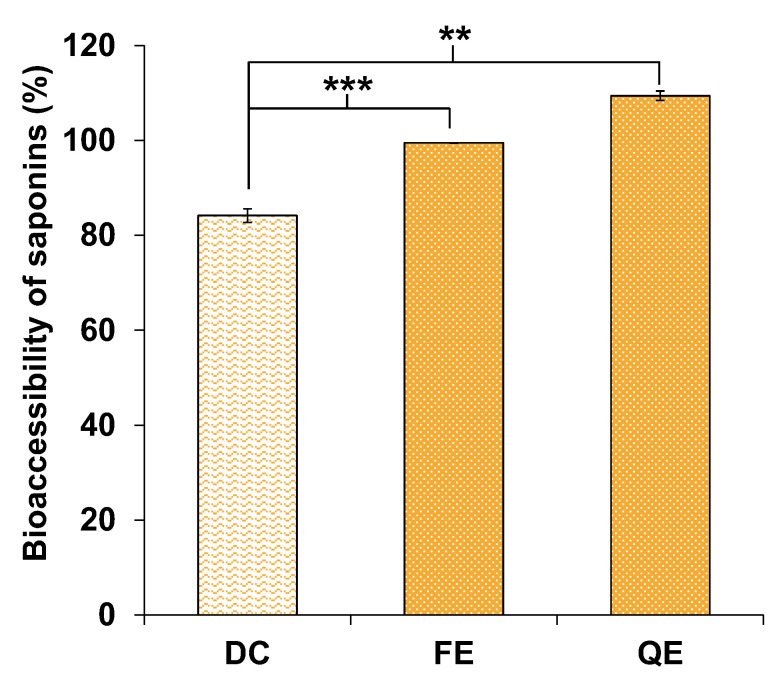
Bioaccessibility (%) of the standard dioscin (DC), fenugreek saponins (FE) and quinoa saponins (QE) after their in vitro gastrointestinal digestion. Mean values of FE and QE are significantly different to DC if *p* ≤ 0.01 (**) or *p* ≤ 0.001 (***).

After the in vitro gastrointestinal digestion of FE and QE, the bioaccessibility of total saponins from each extract was determined by HPLC-DAD, as shown in Figure 2. The saponins from both FE and QE were completely bioaccessible, meaning that the entire saponin fraction was adequately solubilized and dispersed in the micellar phase and no precipitation of the compounds occurred. 

In order to understand if the high bioaccessibilities observed were either a consequence of the amphiphilic nature of saponins or the dispersion-enhancer effect of other compounds contained in the extracts, the gastrointestinal digestion of dioscin (DC), a commercial steroid saponin similar to those found in fenugreek, was performed. Only a standard of steroid saponin was studied because the commercial standard of triterpenoid saponins (hederacoside C) similar to those detected in quinoa co-eluted with digestion components. Interestingly, around 84% of DC was bioaccessible, but had a significantly lower bioaccessibility than saponins from FE (*p* < 0.001) and QE (*p* = 0.002), suggesting that the superior bioaccessibility observed for saponins in the extracts may be achieved when contained in the extracts. This outcome of co-extracted enhancer compounds has been previously confirmed in ethanolic and aqueous ethanolic extracts obtained from fenugreek, quinoa and other seeds, in which the bioaccessibility of saponins, spectrophotometrically determined, positively correlated with both the fat content and the saponin/fat ratio, but negatively correlated with the phenolic content [15]. In such study, we evidenced that a ratio of saponins/fat superior to 1 caused a poor bioaccessibility (<40%), whereas those samples with a ratio lower than 1 showed bioaccessibilities >60%. In the current study, in the case of QE, its saponin/total fat content ratio was estimated to be around 0.15, which would be in agreement with an optimal value for the high bioaccessibility of saponins. However, in the case of FE, the saponin/fat ratio was estimated to be >3 and, hence, a low bioaccessibility of saponins would be expected. As this was not the outcome, its possible phenolic content, which has been suggested as a bioaccessibility-limiting component, and the fact that such extract was obtained by a different procedure than our previous study [15], may be explaining the high bioaccessibility of saponins from FE. In addition, as nearly 50% of the FE remains uncharacterized (Figure 1A), other compounds that have not been identified yet may be enhancing the bioaccessibility of the saponins. 

In any case, the reported bioaccessibilities in this work are in agreement with other values described in the scarce literature. Triterpenoid saponins from chickpea and soy contained in bread have shown high bioaccessibilities (above 85%) after in vitro digestion [49]. In addition, the stability of saponins, but not the bioaccessibility, has been recently assessed with steroid saponins from asparagus and recoveries between 89 and 94% were achieved, although these values refer to the amount of saponins in the whole digestion medium and not in the micellar phase [50].

Therefore, the obtained evidences suggest that saponins contained in saponin-rich extracts from fenugreek and quinoa seeds have a high bioaccessibility, which is likely favoured by the co-existence of other compounds in the extracts. Nevertheless, further studies should be considered in which more digestion replicates are performed, in order to confirm the bioaccessibility values observed for DC and saponins from fenugreek and quinoa.

#### 3.4.2. Sapogenins

The bioaccessibility of the sapogenins contained in HFE and HQE was assessed by GC-MS-FID previous trimethylsilylation of the compounds extracted from both the digestion medium and the micellar phase. 

**Figure 3:**
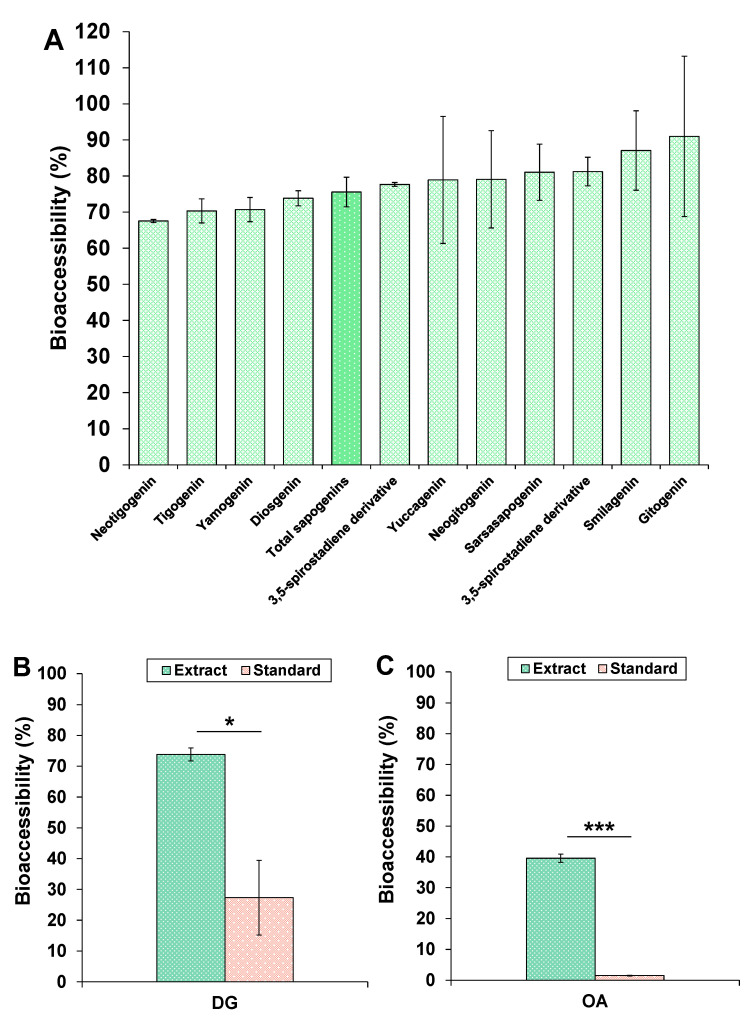
Bioaccessibility (%) of (**A**) steroidal sapogenins contained in HFE, (**B**) diosgenin (DG) from standard and from HFE, and (**C**) oleanolic acid (OA) from standard and from HQE after their in vitro gastrointestinal digestion. Mean values are significantly different to the standard if *p* ≤ 0.05 (*) and *p* ≤ 0.001 (***).

The bioaccessibilities of the eleven identified steroidal sapogenins in the HFE are shown in Figure 3A. In general terms, it can be observed that all sapogenins exhibited good bioaccessibilities, which were above 70%. Diosgenin, the most abundant sapogenin in the extract and the most studied aglycone from fenugreek, presented a good bioaccessibility (around 74%), which was very similar to that of its epimer yamogenin (close to 70%). Two aglycones, smilagenin and gitogenin, appeared to have bioaccessibilities around 90%. Considering the sum of total sapogenins it was estimated that total steroidal sapogenins from fenugreek exhibited a bioaccessibility of 76%. Therefore, despite the fact that the bioaccessibility of sapogenins from HFE was high, such value was lower than that of saponins (Figure 2), being in agreement with the general idea about the better bioaccessibility of saponins respect to sapogenins. 

As performed with saponins, the evaluation of the bioaccessibility of a commercial standard of diosgenin (DG) was assessed in order to understand to what extent would be these steroidal sapogenins bioaccessible when digested alone (Figure 3B). Interestingly, it was found that the DG standard exhibited a low bioaccessibility (27%) when compared to this same compound contained in the HFE (*p* = 0.03), which confirmed the fact that other components of the extract were actively enhancing the solubility and dispersion of DG and other steroidal aglycones in the digestion medium. In this sense, pentacyclic triterpenes from a *Calendula officinalis* extract also showed enhanced bioaccessibilities when co-digested with olive oil, which was explained by an increase in the micellar surface due to lipid digestion products, as FFA and partial glycerides, compared to the absence of oil. This favoured the formation of available micellar structures for inclusion of hydrophobic compounds present in the aqueous media [25]. The solubility-enhancement effects of lipid products have been also recently proposed as a mechanism that assures a high intestinal permeability of DG [27]. Therefore, in the current study, the high lipid content of the HFE under the form of FFA and partial glycerides (Table 2 and Figure 1A) might act as solubility-enhancer by increasing the micellar surface for hydrophobic DG to be included and dispersed in the aqueous media. To the best of our knowledge, this is the first work that has assessed the bioaccessibility of diosgenin and other structurally similar steroidal sapogenins.

Regarding the bioaccessibility of the triterpenoid sapogenins in the HQE, only oleanolic acid (OA) was detected by the analytical tool employed, since the rest of sapogenins were not detected neither in the micellar phase nor in the whole digestion media. The bioaccessibilities of OA after digestion of both its own standard and the HQE are shown in Figure 3C. Similar to what occurred to DG, significant differences were found for OA when digested alone or when contained in the extract (*p* < 0.001). While the standard exhibited an extremely poor bioaccessibility (lower than 2%), likely due to its poor solubility, the compound contained in the HQE showed an intermediate bioaccessibility close to 40%.

Very recently, Zhao et al. [24] described that the bioaccessibility of OA contained in a methanolic extract from *Crataegus pinnatifida* was almost identical (38.4%) to the bioaccessible fraction of OA in this work. Therefore, even though the bioaccessibility of OA in the present work was not as high as the values described for the sapogenins in the HFE, it is evidenced once again the bioaccessibility-enhancing properties of the co-extracted components in the extract, probably lipid components, given that 80% of the HQE accounted for FFA, MAG and DAG. Hence, both results show the relevance of considering the role of co-extracted compounds in the improvement of the bioaccessibility of target bioactive molecules from natural extracts, such as sapogenins. Additionally, these results also suggest the possible interest of assessing the effect of the co-digestion of this and similar extracts with oily “excipient foods” such as healthy or functional vegetable oils [25,51], considering that acceptable bioaccessibilities may be achieved without the need of developing complex and costly formulations for the oral delivery of lipophilic compounds, as sapogenins might be. Nevertheless, in order to confirm the different bioaccessibility values observed for the different sapogenins detected, a larger amount of digestion replicates should be performed in further works.

#### 3.4.3. Other Minor Bioactive Compounds of the Extracts

Taking into account the described composition of the hydrolyzed extracts in other bioactive compounds together with sapogenins (Figure 1), the evaluation of the bioaccessibility of such molecules in the HFE and HQE was considered of interest in order to deepen into the full potential of these extracts. Nevertheless, it was necessary to conduct a selection of compounds due to the existence of chromatographic co-elutions with other components from the digestion medium itself. 

**Figure 4:**
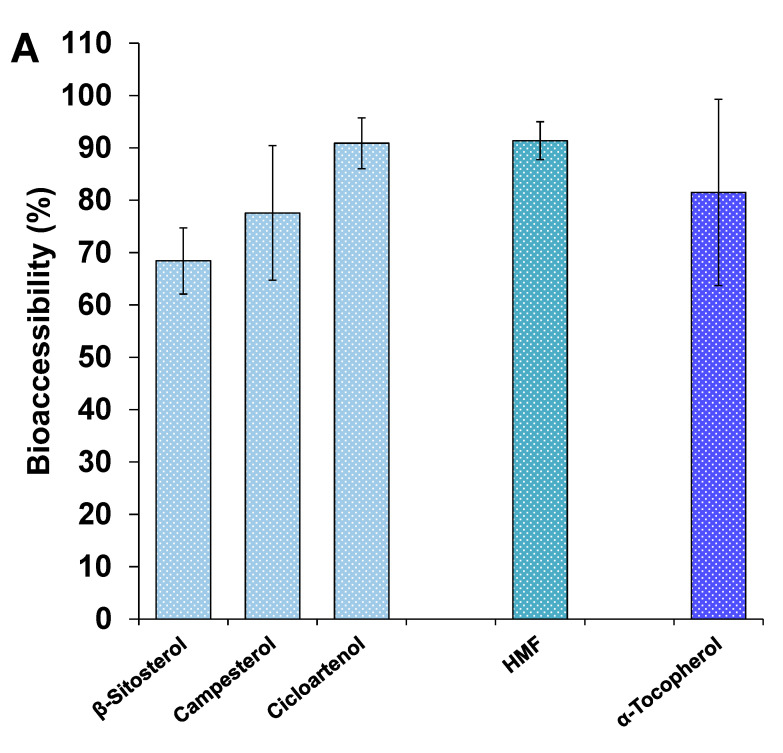

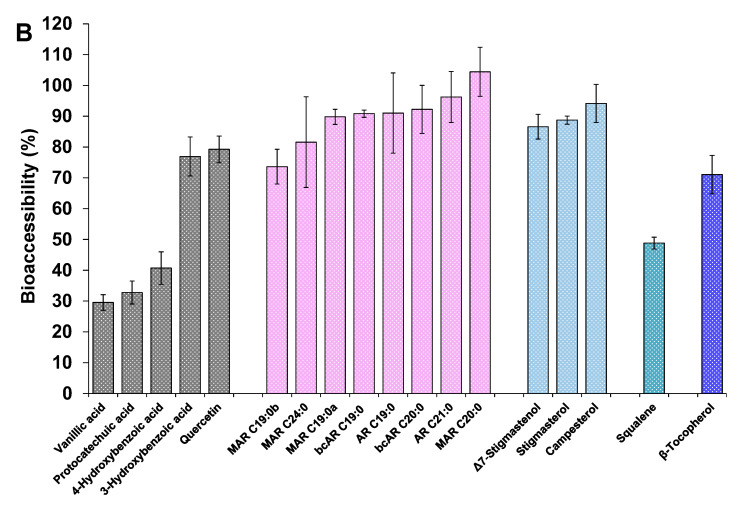
Bioaccessibility (%) of other bioactive compounds from (**A**) HFE and (**B**) HQE after their in vitro gastrointestinal digestion.

Regarding HFE, the bioaccessibility of phytosterols, it ranged between 68 and 90% (Figure 4A). The bioaccessibility of this whole family of compounds was calculated to be close to 70%, meaning that these compounds might effectively disperse in the aqueous media and be included in micellar structures, which would allow to exert their main bioactive effect as cholesterol-reducing agents in the intestinal aqueous media [52]. In fact, in a very recent work performed by ourselves (currently under review, for being published elsewhere), we demonstrated this potential hypocholesterolemic effect of the hydrolyzed extracts from fenugreek and quinoa, which was positively correlated with the phytosterol content of the extracts. The high bioaccessibility observed for phytosterols in the current study would hence support the idea that these compounds were actively participating in the observed displacement of cholesterol from the mixed micelles in such study. 

The bioactive compound α-tocopherol was also detected in the digested samples of HFE and its bioaccessibility was high (around 80%, Figure 4A). It has been proposed that the bioaccessibility of vitamin E is quite variable, being highly dependent on the food matrix in which it is contained. However, Nagao et al. [53] found consistent high bioaccessibilites of α-tocopherol (around 80%) when contained in different vegetables such as pumpkin and carrot, with no significant effects of soybean oil when co-digested. 

Finally, the bioaccessibility of 5-hydroxymethylfurfural (HMF) was also assessed. The previously described duality of this compound, either as potentially toxic and/or bioactive, motivated the inclusion of this compound in the bioaccessibility analysis. Nearly all the HMF contained in the HFE was bioaccessible (around 90%) and, in fact, out of all the compounds studied in this extract, HMF was the one with the highest bioaccessibility, probably due to its easy solubilization in the aqueous media. Quite variable values of bioaccessibility for HMF contained in different dried fruits have been described, being as high as 95% for berries and as low as 8% for plums [54]. In any case, in the current study, it can be confirmed that this compound contained in the HFE would be highly available for its potential intestinal absorption, though its toxicity/bioactivity when contained in the hydrolyzed extracts would need to be further elucidated. 

With regard to the bioaccessibility of bioactives in HQE (Figure 4B), 18 compounds were detected in the digested samples, being mainly phenolic compounds, ARs and phytosterols. The bioaccessibilities of phenolic compounds were quite variable, since those from vanillic acid, protocatechuic acid and 4-hydroxybenzoic acid were moderate in general (between 30–40%), but those from 3-hydroxybenzoic acid and quercetin were considerably higher (close to 80%). It is worth mentioning that quercetin, a flavonoid with multiple health-related bioactivities, has been generally considered to have a very poor water solubility and bioavailability [55]. In fact, Chen et al [56] recently demonstrated an increase in the bioaccessibility of quercetin from around 10% in an aqueous solution to as high as 74% in an excipient emulsion stabilized by casein. Here, we demonstrated that the hydrolysis process of saponin-rich extracts from quinoa might be an efficient and inexpensive process for obtaining extracts whose particular composition, probably lipid compounds, guarantees a high bioaccessibility for other co-extracted poor water soluble compounds, such as quercetin. 

Regarding the bioaccessibility of ARs, all eight identified phenolic lipids showed high bioaccessibilities, which ranged between 70 and 100% (Figure 4B). The elevated lipophilicity of these compounds suggests that very low bioaccessibilities in the intestinal aqueous medium would be expected from ARs. However, it was observed the opposite outcome, suggesting again that the lipophilic nature of the HQE assures in general very adequate bioaccessibilities of poor soluble compounds, including ARs. As far as we are aware, this is the first work that has described the bioaccessibility of ARs, including bcARs and MARs. In this sense, Ross et al. [45] confirmed the detection of some ARs in fasting human plasma, suggesting that these compounds are, to some extent, bioavailable. Further studies assessing the bioaccessibility of ARs standards or their isolates might be worth to be explored in order to deeply understand the behaviour of these compounds in the gastrointestinal tract. 

Regarding phytosterols, and similar to what occurred in the HFE, all three identified compounds were very highly bioaccessible, which confirmed again that the previously demonstrated cholesterol-reducing effects of HQE (under review, to be published elsewhere) are mostly due to the adequate dispersion of phytosterols in the aqueous micellar phase. 

The bioactive β-tocopherol was detected in the digested samples of HQE and its bioaccessibility was high (around 70%), although slightly lower than its α form in the HFE. Also, among all the bioactives detected in the HQE, squalene exhibited the lowest bioaccessibility (close to 50%). Alberdi-Cedeño et al. [57] have very recently determined that the bioaccessibility of squalene contained in olive oil was complete. 

To sum up, considering the total of each of the families studied and regardless of the extract, their bioaccessibility decreased in the following order: triterpenoid saponins > steroid saponins, ARs > phytosterols from HQE > α-tocopherol from HFE > steroid sapogenins > phytosterols from HFE > β-tocopherol from HQE > phenolic compounds > squalene > triterpenoid sapogenins. With the purpose of confirming all these bioaccessibilities from a great amount of different compounds contained in the hydrolyzed extracts, a larger amount of digestion replicates should be performed in further works. In any case, this study evidences that the different bioactive co-existing compounds of natural extracts have a diversity of bioaccessibilities which, although high for most of compounds, should also be considered in order to fully understand their final potential bioactivity.

## Figures and Tables

**Table 1:** Saponin and sapogenin content (g/100 g) of extracts from fenugreek (FE) and quinoa (QE) and their hydrolyzed extracts (HFE and HQE) *.

Rt (Min)	Compound	FE	HFE
	**SAPONINS**	**31.13**	**2.01**
12.78	Fenugreek saponin I	6.63	n.d.
12.87	Fenugreek saponin II	10.66	n.d.
13.09	Fenugreek saponin III	6.02	0.64
14.17	Fenugreek saponin IV	5.02	1.05
14.75	Fenugreek saponin V	2.80	0.33
	**SAPOGENINS**		**8.14**
20.80	3,5-Spirostadiene derivative	n.d.	0.43
20.90	3,5-Spirostadiene derivative	n.d.	0.13
21.66	Smilagenin	n.d.	0.24
21.79	Sarsasapogenin	n.d.	0.64
22.24	Diosgenin	n.d.	2.57
22.34	Tigogenin	n.d.	0.60
22.39	Yamogenin	n.d.	1.23
22.48	Neotigogenin	n.d.	0.64
23.52	Yuccagenin	n.d.	0.03
23.70	Gitogenin	n.d.	1.21
23.88	Neogitogenin	n.d.	0.42
		**QE**	**HQE**
	**SAPONINS**	**7.86**	**2.00**
13.44	Quinoa saponin I	1.29	0.23
13.75	Quinoa saponin II	3.74	1.39
14.38	Quinoa saponin III	2.82	0.38
	**SAPOGENINS**		**5.63**
24.25	Oleanolic acid	n.d.	1.55
25.24	Hederagenin	n.d.	0.82
27.01	Serjanic acid	n.d.	1.83
28.47	Phytolaccagenic acid	n.d.	1.44

n.d. = not detected, * The saponin content refers to the amount of saponins in a single extract derived from the mixture of at least five replicate extractions. The sapogenin content refers to the amount in a single extract derived from the mixture of at least three replicate hydrolysis.

**Table 2:** GC-MS Characterization of Extracts from Fenugreek (*Trigonella foenum-graecum* L.) *.

Rt (Min)	Compound	FE		HFE	
	**LIPIDS**	**Area**	**g/100 g**	**Area**	**g/100 g**
	**Fatty acids**		**2.30**		**35.21**
12.69	Fatty acid n.i.	n.d.	n.d.	327,178	0.13
13.76	Azelaic acid	n.d.	n.d.	976,973	n.q.
14.09	Tetradecanoic acid	n.d.	n.d.	335,623	0.14
14.74	*n*-Pentadecanoic acid	n.d.	n.d.	643,202	0.18
15.43	Palmitic acid	2,556,971	0.47	42,547,201	6.50
15.84	*cis*-10-Heptadecenoic acid	n.d.	n.d.	873,397	0.22
15.97	Heptadecanoic acid	n.d.	n.d.	1,616,500	0.33
16.49	Linoleic acid + oleic acid	8,443,651	1.44	165,223,256	25.00
16.60	Stearic acid	1,976,029	0.38	10,112,280	1.61
17.41	α-Linolenic acid	n.d.	n.d.	1,386,960	0.29
17.50	Fatty acid n.i.	n.d.	n.d.	578,427	0.17
17.63	Arachidic acid	n.d.	n.d.	780,182	0.20
18.64	Docosanoic acid	n.d.	n.d.	498,225	0.16
19.11	Fatty acid n.i.	n.d.	n.d.	414,082	0.15
19.57	Tetracosanoic acid	n.d.	n.d.	244,503	0.12
	**Glycerides**		**0.38**		**7.08**
18.26	2-Monopalmitin	n.d.	n.d.	312,684	0.09
18.43	1-Monopalmitin	660,615	0.22	749,955	0.25
19.08	2-Monoolein	n.d.	n.d.	519,155	0.17
19.25	1-Monoolein	n.d.	n.d.	1,333,086	0.45
19.35	2-Monostearin	493,752	0.16	236,519	0.06
31.22	Diglyceride n.i.	n.d.	n.d.	326,190	1.32
32.15	Diglyceride n.i.	n.d.	n.d.	495,677	1.79
35.98	Diglyceride n.i.	n.d.	n.d.	305,674	1.27
37.32	Diglyceride n.i.	n.d.	n.d.	453,921	1.67
	**NITROGEN COMPOUNDS**				
	**Amino acids and derivatives**		**5.96**		
9.08	l-Valine	74,446	0.02	n.d.	n.d.
11.59	Glutamic acid derivative n.i.	1,157,128	0.27	n.d.	n.d.
11.67	Glutamic acid	21,256,096	4.97	n.d.	n.d.
11.77	Glutamic acid derivative n.i.	1,508,906	0.35	n.d.	n.d.
11.83	Glutamic acid derivative n.i.	705,448	0.17	n.d.	n.d.
12.58	Phenylalanine	485,697	0.11	n.d.	n.d.
14.78	l-Tyrosine	283,205	0.07	n.d.	n.d.
	**Nitrogen compounds, non-protein**				
18.75	Adenosine	252,644	n.q.	n.d.	n.d.
	**PHENOLIC COMPOUNDS**				
12.56	4-Hydroxybenzoic acid	n.d.	n.d.	320,566	0.03
12.56	Phenolic compound n.i.	599,743	0.05	n.d.	n.d.
	**ORGANIC ACIDS**				
8.27	Levulinic acid	n.d.	n.d.	1,064,940	n.q.
12.40	β-Hydroxy-β-methylglutaric acid	n.d.	n.d.	288,134	n.q.
	**CARBOHYDRATES AND DERIVATIVES**				
	**Sugars**		**1.73**		
16.55	Monosaccharide n.i.	2,779,858	0.09	n.d.	n.d.
18.93	Sucrose	5,189,844	1.09	n.d.	n.d.
19.30	Disaccharide n.i.	1,134,911	0.24	n.d.	n.d.
19.53	Disaccharide n.i.	1,129,958	0.24	n.d.	n.d.
19.90	Disaccharide n.i.	199,114	0.04	n.d.	n.d.
21.24	Disaccharide n.i.	88,523	0.03	n.d.	n.d.
	**Sugar alcohols**		**1.70**		
14.18	Sugar alcohol n.i.	2,851,411	1.55	n.d.	n.d.
15.85	Sugar alcohol n.i.	279,870	0.15	n.d.	n.d.
	**TOCOLS**				
21.07	α-Tocopherol	122,692	0.10	330,911	0.26
	**PHYTOSTEROLS**		**0.82**		**1.86**
21.81	Campesterol	152,569	0.12	332,792	0.26
22.38	β-Sitosterol	673,182	0.49	2,230,012	1.31
22.85	Cycloartenol	262,446	0.21	382,728	0.29
	**OTHER ORGANIC COMPOUNDS**				
9.96	5-(Hydroxymethyl)furfural	n.d.	n.d.	794,089	n.q.
	**NON-IDENTIFIED COMPOUNDS**				
9.53	n.i.	n.d.	n.d.	272,457	n.d.
9.96	n.i.	379,413	n.d.	n.d.	n.d.
10.07	n.i.	640,401	n.d.	n.d.	n.d.
15.64	n.i.	679,227	n.d.	n.d.	n.d.
15.93	n.i.	613,602	n.d.	n.d.	n.d.
16.95	n.i.	1,047,810	n.d.	n.d.	n.d.

n.q. = not quantitated; n.i. = not identified; n.d. = not detected. * The content of each compound refers to their amount in a single extract derived from the mixture of at least five replicate extractions (FE) or three replicate hydrolysis (HFE).

**Table 3:** GC-MS Characterization of Extracts from Quinoa (*Chenopodium quinoa* Willd.) *.

Rt (Min)	Compound	QE		HQE	
	**LIPIDS**	**Area**	**g/100 g**	**Area**	**g/100 g**
	**Fatty acids**		**18.67**		**57.37**
10.81	Fatty acid n.i.	450,537	0.15	105,632	0.10
13.74	Azelaic acid	295,429	n.q.	624,302	n.q.
14.09	Tetradecanoic acid	n.d.	n.d.	967,829	0.23
14.60	Palmitic acid, methyl ester	n.d.	n.d.	564,801	0.17
14.74	*n*-Pentadecanoic acid	n.d.	n.d.	436,759	0.15
15.24	Palmitoleic acid	n.d.	n.d.	1,594,816	0.33
15.40	Palmitic acid	21,585,329	2.57	63,448,504	9.65
15.67	Linoleic acid methyl ester	709,965	0.19	1,460,352	0.31
15.79	Heptadecanoic acid	645,089	0.18	n.d.	n.d.
16.46	Linoleic acid + Oleic acid	112,939,760	13.82	292,989,296	44.26
16.57	Stearic acid	2,598,181	0.48	5,188,940	0.87
16.80	*cis*-10-Nonadecenoic acid	573,992	0.17	1,192,260	0.27
16.95	Nonadecanoic acid	717,759	0.19	n.d.	n.d.
17.11	Branched chain fatty acid n.i.	558,659	0.17	n.d.	n.d.
17.49	Fatty acid n.i.	1,977,879	0.38	2,625,371	0.48
17.62	Eicosanoic acid	388,780	0.14	664,297	0.19
18.52	*cis*-13-Docosenoic acid	834,878	0.21	1,910,933	0.37
	**Glycerides**		**23.39**		**23.15**
18.26	2-Monopalmitin	n.d.	n.d.	1,026,164	0.35
18.43	1-Monopalmitin	2,554,881	0.85	2,241,608	0.75
18.64	Monoglyceride n.i.	n.d.	n.d.	2,122,285	0.71
19.08	2-Monoolein	n.d.	n.d.	1,797,832	0.61
19.26	1-Monoolein	n.d.	n.d.	5,789,486	1.81
19.25	Monoglyceride n.i.	2,419,864	0.81	625,561	0.21
19.36	2-Monostearin	1,504,555	0.51	885,292	0.30
31.22	Diglyceride n.i.	1,711,567	5.09	892,885	2.87
32.15	Diglyceride n.i.	1,210,341	3.73	1,589,691	4.76
35.98	Diglyceride n.i.	1,895,510	5.59	1,322,477	4.03
37.33	Diglyceride n.i.	2,338,224	6.80	2,321,607	6.75
	**NITROGEN COMPOUNDS**				
	**Amino acids and derivatives**		**1.07**		
9.09	l-Valine	520,768	0.12	n.d.	n.d.
9.65	l-Leucine	593,774	0.14	n.d.	n.d.
9.82	l-Isoleucine	573,058	0.13	n.d.	n.d.
9.84	l-Proline	310,659	0.07	n.d.	n.d.
12.58	Phenylalanine	755,735	0.18	n.d.	n.d.
14.79	l-Tyrosine	1,841,249	0.43	n.d.	n.d.
	**Nitrogen compounds, non-protein**				
18.76	Adenosine	859,568	n.q	n.d.	n.d.
19.03	Nucleoside n.i.	2,105,901	n.q	n.d.	n.d.
	**PHENOLIC COMPOUNDS**		**0.49**		**1.20**
11.91	Phenolic compound n.i.	301,007	0.03	352,057	0.03
12.50	3-Hydroxybenzoic acid	277,150	0.02	228,709	0.02
12.55	4-Hydroxybenzoic acid	316,918	0.03	742,472	0.06
13.56	Vanillic acid	658,070	0.06	2,287,118	0.19
13.97	Protocatechuic acid	1,574,802	0.13	1,100,106	0.09
15.70	Isoferulic acid	770,448	0.07	1,178,230	0.10
15.82	Phenolic compound n.i.	n.d.	n.d.	4,209,075	0.36
21.39	Quercetin	529,905	0.16	1,889,147	0.35
	**ORGANIC ACIDS**				
8.31	Levulinic acid	n.d.	n.d.	6,678,331	n.q.
	**CARBOHYDRATES AND DERIVATIVES**				
	**Sugars**		**15.04**		
13.50	Monosaccharide n.i.	334,468	0.05	n.d.	n.d.
14.02	Monosaccharide n.i.	800,569	0.11	n.d.	n.d.
14.08	Monosaccharide n.i.	869,500	0.12	n.d.	n.d.
14.57	Monosaccharide n.i.	1,341,584	0.18	n.d.	n.d.
15.13	Monosaccharide n.i.	1,725,857	0.23	n.d.	n.d.
17.32	Monosaccharide n.i.	621,039	0.08	n.d.	n.d.
18.16	Monosaccharide n.i.	608,507	0.08	n.d.	n.d.
18.91	Disaccharide n.i.	761,289	1.67	n.d.	n.d.
18.99	Sucrose	70,842,522	12.52	n.d.	n.d.
	**Sugar alcohols**		**0.88**		
13.37	Sugar alcohol n.i.	219,799	0.12	n.d.	n.d.
13.41	Sugar alcohol n.i.	310,261	0.17	n.d.	n.d.
14.89	Sugar alcohol n.i.	1,089,958	0.59	n.d.	n.d.
	**TOCOLS**				
20.38	β-Tocopherol	710,062	0.51	987,442	0.68
21.09	α-Tocopherol + Branched chain alkylresorcinol C20:0	504,040	n.q.	494,045	n.q.
	**ALKYLRESORCINOLS ^1^**		**1.79**		**1.04**
20.57	Branched chain alkylresorcinol C19:0	301,095	0.10	131,551	0.04
20.73	Alkylresorcinol C19:0	157,677	0.05	91,436	0.03
20.86	Methyl alkylresorcinol C19:0 a	162,491	0.05	174,353	0.06
21.04	Methyl alkylresorcinol C19:0 b	89,956	0.03	105,698	0.03
21.22	Alkylresorcinol C20:0	63,163	0.02	66,606	0.02
21.44	Methyl alkylresorcinol C20:0	465,052	0.15	713,693	n.q.
21.56	Branched chain alkylresorcinol C21:0	632,613	0.21	341,593	0.11
21.66	Alkenylresorcinol C21:1	345,020	0.11	199,108	0.07
21.77	Alkylresorcinol C21:0	404,519	0.13	198,851	0.07
21.94	Methyl alkylresorcinol C21:0 a	363,033	0.12	355,126	0.12
22.05	Methyl alkenylresorcinol C21:1	89,517	0.03	84,248	0.03
22.10	Alkenylresorcinol C22:1	44,641	0.01	n.d.	n.d.
22.18	Methyl alkylresorcinol C21:0 b	135,872	0.04	198,437	0.07
22.24	Branched chain alkylresorcinol C22:0	588,653	0.19	376,310	0.12
22.53	Methyl alkenylresorcinol C22:1 + β-Amyrin	111,487	n.q.	157,275	n.q.
22.69	Methyl alkylresorcinol C22:0	372,702	0.12	327,208	0.11
22.84	Branched chain alkylresorcinol C23:0 a	294,763	0.10	n.d.	n.d.
22.99	Alkenylresorcinol C23:1	115,112	0.04	48,642	0.02
23.38	Methyl alkylresorcinol C23:0 a	185,482	0.06	165,900	0.05
23.77	Branched chain alkylresorcinol C24:0	238,231	0.08	93,178	0.03
24.41	Methyl alkylresorcinol C24:0	145,075	0.05	105,472	0.03
24.60	Branched chain alkylresorcinol C25:0	149,488	0.05	44,779	0.01
25.35	Methyl alkylresorcinol C25:0	41,486	0.01	34,293	0.01
25.89	Branched chain alkylresorcinol C26:0	43,240	0.01	n.d.	n.d.
	**PHYTOSTEROLS**		**1.47**		**2.35**
21.82	Campesterol	n.d.	n.d.	93,024	0.08
21.99	Stigmasterol	129,590	0.10	270,847	0.21
22.39	β-Sitosterol	847,328	0.60	1,629,923	1.02
22.81	Δ7-Stigmastenol	1,143,706	0.77	1,663,885	1.04
	**OTHER ORGANIC COMPOUNDS**				
9.96	5-(Hydroxymethyl)furfural	n.d.	n.d.	4,142,040	n.q.
19.56	Squalene	2,151,321	0.97	3,244,348	1.40
	**NON-IDENTIFIED COMPOUNDS**				
10.68	n.i.	n.d.	n.d.	145,243	n.q.
11.87	n.i.	530,418	n.q.	n.d.	n.d.
13.11	n.i.	199,292	n.q.	n.d.	n.d.
13.61	n.i.	387,038	n.q.	n.d.	n.d.
14.53	n.i.	5,992,880	n.q.	n.d.	n.d.
14.68	n.i.	4,571,935	n.q.	n.d.	n.d.
16.57	n.i.	3,652,082	n.q.	n.d.	n.d.
17.59	n.i.	n.d.	n.d.	731,295	n.q.

^1^ MARs with a methyl group at the n-2 carbon have been labelled with an “a” and those with a methyl group at the n-3 carbon with a “b”. In bcARs, such categorization corresponds to a different position of a methyl group along the alkyl chain, as proposed by Ross et al. [45]. n.q. = not quantitated; n.i. = not identified; n.d. = not detected. * The content of each compound refers to their amount in a single extract derived from the mixture of at least five replicate extractions (QE) or three replicate hydrolysis (HQE).

## Supplementary Materials

The following are available online at https://www.mdpi.com/2304-8158/9/9/1159/s1, Figure S1: (A) HPLC-DAD chromatogram showing the identified saponins in FE and (B) GC-MS chromatogram showing the identified sapogenins in HFE. Figure S2: (A) HPLC-DAD chromatogram showing the identified saponins in QE and (B) GC-MS chromatogram showing the identified sapogenins in HQE.
