# Supplementary material for: Chemical Characterization and Bioaccessibility of Bioactive Compounds from Saponin-Rich Extracts and Their Acid-Hydrolysates Obtained from Fenugreek and Quinoa
Source: Foods. 2020 Aug 21;9(9):1159. doi: 10.3390/foods9091159 (PMC7555840; doi:10.3390/foods9091159)

Supplementary material

Figure S1. A) HPLC-DAD chromatogram showing the identified saponins in FE and B) GC-MS chromatogram showing the identified sapogenins in HFE.

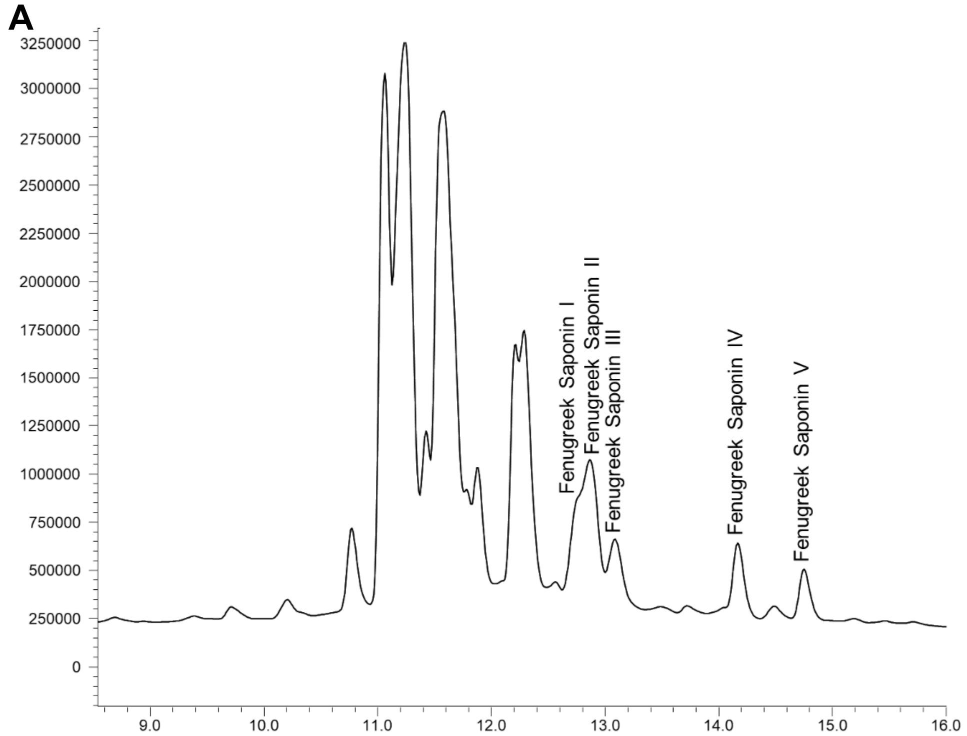

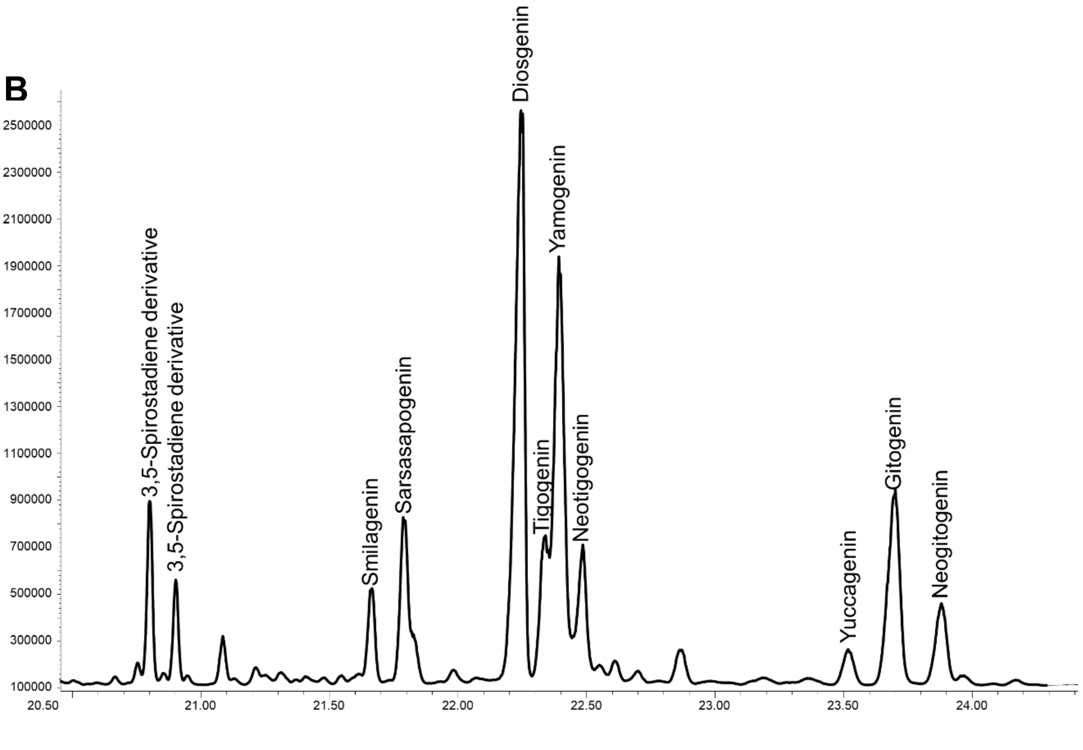

Figure S2. A) HPLC-DAD chromatogram showing the identified saponins in QE and B) GC-MS chromatogram showing the identified sapogenins in HQE.

A

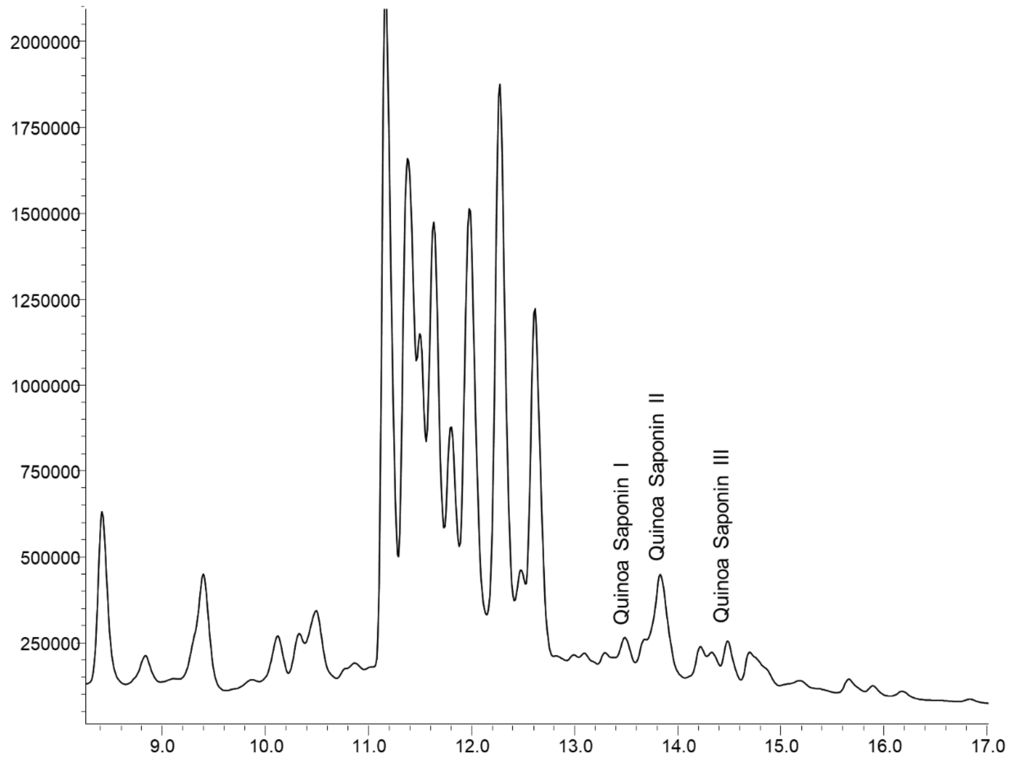

B

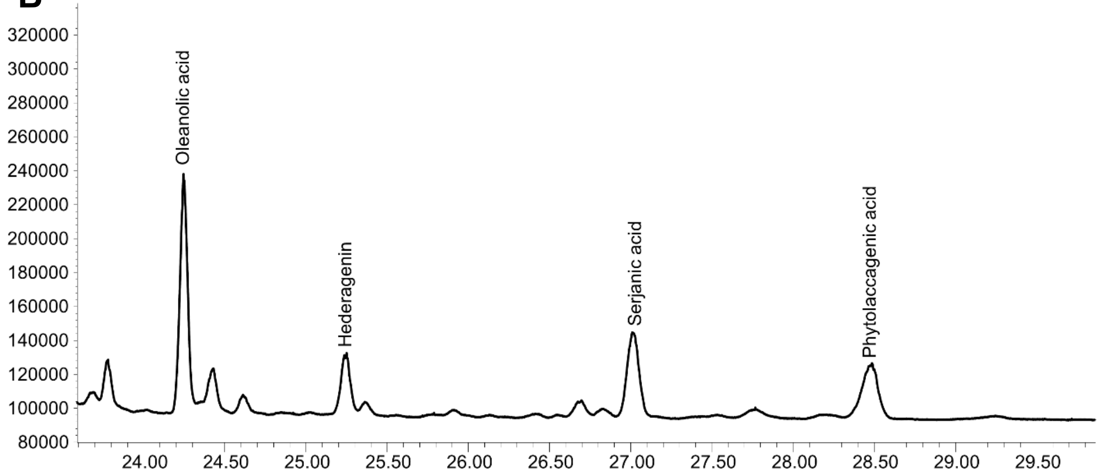

Supplement: Supplementary file 1 [file foods-09-01159-s001.pdf]
